# A Small-Molecule TrkB Agonist Promotes Synaptic Resilience Against Aβ and Tau Oligomers and Mitigates Alterations in TrkB-Akt and Fyn Signaling

**DOI:** 10.3390/cells15191822

**Published:** 2026-10-07

**Authors:** Tao Yang, Kevin C. Tran, Albert Leng, Vanessa F. Langness, Stephen M. Massa, Frank M. Longo

**Affiliations:** 1 Department of Neurology and Neurological Sciences, Stanford University, Stanford, CA 94305, USA; yangt@stanford.edu (T.Y.); song.leng@cshs.org (A.L.); vfl@stanford.edu (V.F.L.); 2 Department of Neurology, San Francisco Veterans Affairs Health Care System, San Francisco, CA 94121, USA; 3 Department of Neurology, University of California, San Francisco, CA 94121, USA; 4 Wu Tsai Neurosciences Institute, Stanford University, Stanford, CA 94305, USA

**Keywords:** Akt, amyloid-beta, dendritic spine, Fyn, PTX-BD4-3, resilience, synapse, tau, tropomyosin-receptor kinase B (TrkB)

## Abstract

**Highlights:**

**What are the main findings?**

- In vitro, PTX-BD4-3 protected against oAβ-induced synaptic protein loss and elevation of Fyn, NR2B, and tau phosphorylation. Preservation of synaptic proteins following oAβ exposure was TrkB-dependent. PTX-BD4-3 also protected against oTau-induced dendritic spine loss.
- In APP^L/S^ mice, PTX-BD4-3 preserved synaptic proteins and dendritic spines, improved Y-maze performance, and was associated with increased TrkB–Akt and reduced Fyn–tau signaling.

**What are the implications of the main findings?**

- TrkB partial agonism may enhance synaptic resilience against oAβ- and oTau-associated neurodegeneration.
- These findings support further investigation of PTX-BD4-3 and TrkB partial agonism as potential therapeutic strategies for preserving synaptic function in Alzheimer’s disease.

**Abstract:**

Background: Pathogenic amyloid-beta and tau oligomers (oAβ, oTau) drive synaptic degeneration and cognitive decline in Alzheimer’s disease (AD). Tropomyosin receptor kinase B (TrkB), expressed in neurons and glia, supports synaptic integrity in AD-affected regions. We tested whether PTX-BD4-3, a small-molecule TrkB partial agonist, promotes neuroprotective signaling, reduces degenerative signaling, and prevents synaptic loss in an AD mouse model and hippocampal neurons exposed to oAβ or oTau. Methods: Cultured hippocampal neurons were treated with oAβ or oTau ± PTX-BD4-3. Effects on synaptic proteins (±TrkB-blocking antibody), spine density, and Fyn, NR2B, and tau phosphorylation as well as tau oligomerization were assessed. Two cohorts of APP^L/S^ mice, 5 and 12 months of age, received intraperitoneal PTX-BD4-3 or vehicle for 3 months, followed by assessment of synaptic proteins, dendritic spines, TrkB signaling, and Y-maze analysis. Results: In vitro, PTX-BD4-3 prevented oAβ-induced synaptic marker loss in a TrkB-dependent manner, reduced phosphorylation of Fyn, NR2B, and tau, suppressed oligomeric tau formation, and attenuated oTau-induced spine loss. In APP^L/S^ mice, PTX-BD4-3 mitigated deficient TrkB/Akt signaling, reduced phosphorylation of Fyn and tau, preserved synaptic markers and dendritic spines, and improved Y-maze performance. Conclusions: Small-molecule TrkB partial agonists may normalize TrkB signaling and promote synaptic resilience, supporting their potential as AD therapeutics.

## 1. Introduction

Signaling through the neurotrophin receptor tropomyosin receptor kinase B (TrkB) activates multiple downstream pathways, including phosphatidylinositol-3 kinase (PI3K)/Akt, mitogen-activated protein kinase (MAPK)/extracellular signal-regulated kinases (ERKs), and phospholipase Cγ (PLCγ)/protein kinase C (PKC), which collectively promote neuroprotection and regulate dendritic spine density, synaptic plasticity, and synaptic integrity [1,2,3,4,5,6]. In Alzheimer’s disease (AD), reduced BDNF levels and impaired TrkB signaling have been widely reported and are associated with synaptic degeneration and cognitive decline [7,8,9,10,11]. In addition, genetic variations in the BDNF gene have been associated with increased AD risk, further suggesting that disruption of BDNF/TrkB signaling may contribute to disease pathogenesis [12,13,14,15]. Consistent with this, activation of BDNF/TrkB signaling reduces tau phosphorylation [16], promotes synaptic function [17], and is inversely correlated with neuritic plaques and neurofibrillary tangles [18]. Moreover, it protects against dendritic spine loss, neuronal and synaptic injury, and cognitive dysfunction in AD models [19,20,21,22]. Together, these data suggest that activating BDNF/TrkB signaling may be beneficial for improving neuronal and synaptic resilience to Aβ-induced toxicity.

In addition to effects through canonical Trk signaling pathways, emerging evidence suggests that BDNF/TrkB signaling can also influence Fyn activity [23,24], thereby affecting synaptic signaling pathways critical for memory and learning. Under normal physiologic conditions, Fyn-mediated phosphorylation of NMDAR-NR2B at Y1472 (p-NR2B^Y1472^) stabilizes its localization at the synapse in dendritic spines and enhances responsiveness to the NMDAR ligand glutamate, supporting synaptic plasticity and memory-related processes [25,26,27,28,29,30]. However, in AD, soluble oligomeric Aβ (oAβ) and tau (oTau), which are considered major contributors to synapse loss and cognitive decline [31,32], can aberrantly activate Fyn signaling. Aβ activation of Fyn promotes excess phosphorylation of the NMDA receptor (NMDAR) NR2B subunit at tyrosine 1472 (Y1472) and tau at tyrosine 18 (Y18) [30,33,34,35]. Elevated tau phosphorylation, including at Y18, promotes oligomerization and aggregation into toxic tau species [36,37]. Consistent with this mechanism, human AD brains exhibit elevated Fyn immunoreactivity in subsets of neurons [38], and elevated cortical tau phosphorylation at the Fyn substrate site Y18 (pTau^Y18^) [39]. Additionally, pTau^Y18^ was detected in brain sections from a tauopathy mouse model but was absent in non-transgenic mice [40]. Thus, Aβ-induced alterations in Fyn localization and activation, as well as tau and NR2B phosphorylation, can lead to aberrant glutamate and NMDAR signaling that can promote synaptic and dendritic spine dysfunction [41,42,43]. Together, these findings identify Fyn–tau–NMDAR signaling as a central mediator of oligomer-induced synaptotoxicity in AD.

Despite the promise of targeting BDNF/TrkB signaling, therapeutic application of BDNF is limited by poor blood–brain barrier (BBB) penetration and a short plasma half-life [44,45,46,47]. To overcome these limitations, small-molecule TrkB ligands have been developed [48,49]. Among these small molecules, 7,8-dihydroxyflavone (7,8-DHF) has been the most extensively studied compound in AD models [50,51,52,53,54,55,56,57]. Additional compounds, including flavone derivatives such as 7,8,3′-trihydroxyflavone and the prodrug R13, natural products such as deoxygedunin, and the partial agonist LM22A-4, have been reported to engage TrkB signaling and to confer neuroprotective and cognitive benefits in various neurodegenerative models. However, the relevance of their signaling mechanisms to disease-specific synaptotoxic pathways remains incompletely understood [58,59,60,61,62,63,64]. Prior studies have largely focused on morphological or behavioral outcomes without directly interrogating synaptotoxic signaling pathways, and the role of potential effects on Fyn–tau–NMDAR signaling has not been explored.

PTX-BD4-3 is a novel small molecule derived from the previously identified LM22A-4 [63], which was modified to achieve higher brain-to-plasma ratios and exhibits TrkB partial agonist activity. In previous studies, PTX-BD4-3 dose-dependently promoted cell survival in both NIH-3T3-TrkB cells and primary hippocampal neurons, but did not affect survival in NIH-3T3 cells expressing TrkA, TrkC, or p75 neurotrophin receptor, or in hippocampal neurons exposed to a TrkB-blocking antibody, suggesting that these effects are TrkB-dependent [65]. In vivo, PTX-BD4-3 increased TrkB signaling and neuronal survival in a mouse model of Rett syndrome [65], and improved synaptic function in inhibitory interneurons in mouse models of epilepsy [66]. However, neither PTX-BD4-3 nor any LM22A-4 derivative has been tested in an AD-related preclinical model. Given this gap and the mitigating effects of TrkB activation in other neurodegenerative disease models, we hypothesized that PTX-BD4-3 might normalize synaptotoxic signaling pathways, synaptic structural measures, dendritic spine integrity, and behavior, and we tested this hypothesis in cultured hippocampal neurons exposed to oAβ or oTau and in an APP/amyloid-based mouse model.

## 2. Materials and Methods

### 2.1. AD Mouse Models and Treatment

**Figure 1:**
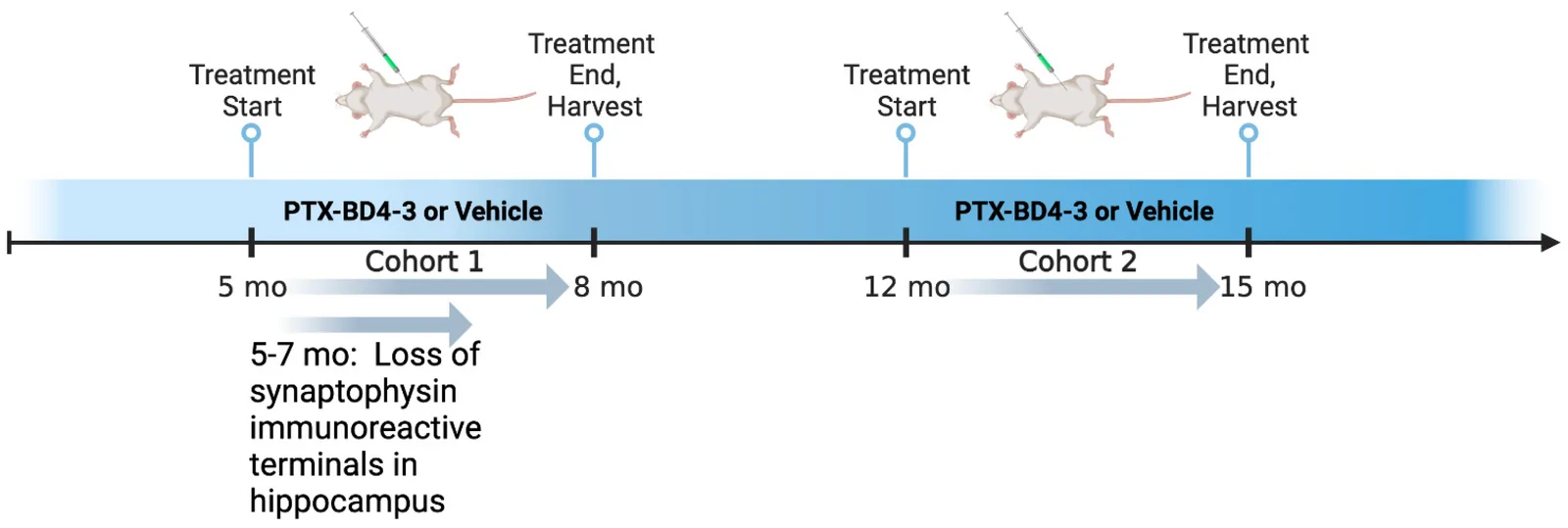
Mouse treatment timeline. Two cohorts of mice were used for mouse experiments: one cohort began treatment at the age of 5 months, and the other cohort began treatment at 12 months. PTX-BD4-3 (50 mg/kg/day) or vehicle was administered intraperitoneally (IP), once daily, 5 days/week for 3 months to WT or APP^L/S^ mice. A final dose of PTX-BD4-3 or vehicle was administered to all the mice 1 h prior to euthanasia and harvest of brain tissue. Created with BioRender.com.

All animal procedures complied with the National Institutes of Health Guide for the Care and Use of Laboratory Animals and were approved by the Institutional Animal Care and Use Committee at Stanford University. The study utilized the transgenic APP London/Swedish (APP^L/S^) mouse line T41B, which overexpresses human amyloid precursor protein 751 (hAPP751) with London (V717I) and Swedish (K670M/N671L) mutations under the murine Thy1 promoter. These mice exhibit synaptophysin reductions by 5–7 months of age in the hippocampus [67,68]. PTX-BD4-3 (benzene-1,3,5-tricarboxylic acid tris- (2-methoxy-ethyl)-methyl-amide) was custom-manufactured by Ricerca Biosciences (Concord, OH, USA). The preparation was characterized by high-performance liquid chromatography and mass spectrometry and had a purity of greater than 97%. For in vivo treatment, PTX-BD4-3 was dissolved in water. The compound was administered intraperitoneally (IP) at a dose of 50 mg/kg/day, 5 days/week for 3 months. Vehicle-treated mice received an equivalent volume of vehicle per weight on the same schedule. Two cohorts of male mice received treatment: one set began treatment at the age of 5 months, and the other set started at 12 months. After 3 months of treatment, a final dose of PTX-BD4-3 was administered to all the mice 1 h before euthanasia (Figure 1). Mice were deeply anesthetized with pentobarbital (40–50 mg/kg, IP) and euthanized by exsanguination, followed by transcranial perfusion with phosphate-buffered saline (PBS). One brain hemisphere was separated and Golgi-stained for spine density analysis; the other hemisphere was dissected to obtain hippocampal tissue for Western blot analysis of signaling proteins, as established in prior studies [69]. The numbers of animals used for each experiment are specified in the figure legends. A total of 114 animals were used in this study.

### 2.2. Modified Golgi Staining, Tracing, and Analysis of Dendrites and Spines

Modified Golgi staining was performed using a previously established protocol [70]. Briefly, after euthanasia, one brain hemisphere from each mouse was quickly excised and immersed in pre-made modified Golgi–Cox staining solution (purchased from Deqiang Jing and Francis Lee, Cornell University). Brain tissues were incubated in the staining solution for 9 days at room temperature in the dark. Then, the brains were transferred to 30% (*w*/*v*) sucrose in distilled H_2_O (dH_2_O) at 4 °C for 72 h, and the solution was changed after the first 12 h. Brains were then sectioned coronally at 150 μm thickness using a vibratome (Leica Microsystems, Wetzlar, Germany). Free-floating sections were carefully mounted onto slides coated with 0.3% gelatin and allowed to adhere completely. Slides were then dipped three times in 40% sucrose and air-dried for 72 h in the dark. After drying, sections were rinsed thoroughly in dH_2_O and developed using the modified Golgi–Cox developing solution according to the established protocol [70]. Following development, sections were washed extensively with dH_2_O, dehydrated through a graded ethanol series (50%, 70%, 95%, and 100%), cleared in xylene, and coverslipped using distyrene, plasticizer, and xylene (DPX) mounting medium (Sigma-Aldrich Corp., St. Louis, MO, USA). Stained sections were allowed to cure completely before imaging and quantitative dendritic spine analysis.

Golgi-stained hippocampal pyramidal neurons, including their dendrites and spines, were traced using Neurolucida software (version 2020.1.1, MBF Bioscience, Williston, VT, USA) with a Zeiss brightfield microscope by an experimenter blinded to genotype/treatment, following our previously established protocol [70,71]. First, the 10× objective was used to identify and focus on the hippocampal CA1 region using previously described anatomical landmarks in coronal sections [72]. We selected neurons for dendritic tracing following Neurolucida protocol guidelines. Specifically, we selected relatively isolated neurons that were free from interference from neighboring dendrites and somas and that displayed intense staining of dendritic arborizations, allowing for unambiguous identification of dendritic spines. After a neuron was selected, the 100× oil immersion objective was used to perform real-time dendritic tracing using Neurolucida’s live mode. The soma was outlined using the ‘Cell Body’ option, followed by manual tracing of apical and basal dendrites. After tracing, spines were manually marked along the dendrites. Spines were defined as protrusions perpendicular to the dendritic shaft, measuring 0.5–2 μm in length with a clear spine neck or head. Dendrite length and number of apical and basal spines for each neuron were calculated using Neurolucida Explorer software (version 2020.3.1, MBF Bioscience, Williston, VT, USA). The apical and basal dendritic spine densities for each neuron were calculated by dividing the number of spines by the apical or basal dendrite length.

### 2.3. Enrichment of Hippocampal Synaptosomes

We modified a previously published method [73] to enrich hippocampal synaptosomes, as described previously [33]. Briefly, mouse hippocampi were treated with ice-cold sucrose buffer (10 mM Tris-HCl, pH 7.4, 320 mM sucrose, 4 mM HEPES, 1 mM Na_3_VO_4_, 1 mM EDTA), sonicated for 10 s, and then incubated on ice for 30 min. The samples were centrifuged at 1000× *g* for 12 min at 4 °C, and the pellets (P1) containing the nuclear/cytoskeletal fraction were discarded. The synaptosome-containing supernatants (S1) were further centrifuged at 25,000× *g* for 30 min at 4 °C. The synaptosome-enriched pellets were then resuspended in lysis buffer (100 mM NaCl, 20 mM HEPES, 1 mM EDTA, 1 mM dithiothreitol, 1.0% Triton, 1 mM Na_3_VO_4_, 1 complete mini EDTA-free protease inhibitor cocktail tablet/10 mL) and stored at −80 °C for future use.

### 2.4. Western Blot Analyses

Synaptosome samples (20–40 μg total protein/lane, depending on which antibody was used for Western blotting, with equal amounts of protein in each lane per given gel) were electrophoresed on a NuPAGE 4–12% Bis-Tris gel from Thermo Fisher Scientific (Waltham, MA, USA), and then transferred to a PVDF membrane. Membranes were blocked with 5% non-fat dried milk in PBS at room temperature for 1 h. Phospho-specific antibodies were used to probe the membranes first, followed by re-probing with antibodies against the total phosphorylated and unphosphorylated forms of the protein. The following primary antibodies were used: p-TrkB^Y705^ (1:1000, Cat#52705, Signalway Antibody, Greenbelt, MA, USA); p-Akt^Ser473^ (1:2000, Cat#4060, Cell Signaling Technology, Beverly, MA, USA), Akt (1:2000, Cat#9272, Cell Signaling Technology), p-ERK^Thr202/Tyr204^ (1:2000, Cat#9101, Cell Signaling Technology), and ERK (1:2000, Cat#9102, Cell Signaling Technology); TrkB (1:3000, Cat#MA5-38028, Thermo Fisher Scientific, Waltham, MA, USA); actin (1:10,000, Cat#A5441, Sigma-Aldrich Corp., St. Louis, MO, USA); PSD95 (1:4000, Cat#200-301-G34, Thermo Fisher Scientific); synaptophysin (1:4000, Cat#ab32127, Abcam, Fremont, CA, USA); p-FynY416 (1:1000, Cat#2101, Cell Signaling Technology); Fyn (1:1000, Cat#SC-434, Santa Cruz Biotechnology, Dallas, TX, USA); pTauY18 (1:1000, Cat#MB588135, MyBioSource, San Diego, CA, USA); HT7 (1:1000, Cat#MN1000, Thermo Fisher Scientific); and GFAP (1:1000, Cat#G-9269, Sigma-Aldrich Corp.). Secondary antibodies conjugated to horseradish peroxidase (HRP), either anti-rabbit IgG (1:10,000, Cat#31460, Thermo Fisher Scientific) or anti-mouse IgG (1:10,000, Cat#P044701-5, DAKO (Agilent), Glostrup, Denmark), were used to facilitate signal detection. After detection with enhanced chemiluminescence (ECL) reagent (GE Healthcare, Sunnyvale, CA, USA), the densities of the immunoreactive bands were measured using UN-SCAN-IT gel software (version 6.1, Silk Scientific Inc., Orem, UT, USA). Western blot quantification was performed by investigators blinded to genotype and treatment group.

### 2.5. Preparation of Synthetic oAβ and Recombinant oTau

The synthetic oAβ preparation was generated following our previously established protocol and contained primarily oligomeric structures as determined by atomic force microscopy (Digital Instruments, Santa Barbara, CA, USA) [74]. Briefly, synthetic human Aβ_1-42_ peptides (Beta-Amyloid [1-42], HFIP, Cat# A-1163-2, rPeptide, Watkinsville, GA, USA) were first resuspended in anhydrous dimethyl sulfoxide (DMSO; Sigma-Aldrich Corp.) to a stock concentration of 5 mM. The stock solution was then diluted to a final concentration of 80 µM in sterile phosphate-buffered saline (PBS), gently mixed, and incubated at 4 °C for 16–24 h to allow the formation of soluble oAβ. Immediately before use, the preparation was gently mixed with neuronal culture medium at the indicated final concentration. Fresh oAβ preparations were generated for each independent experiment.

Recombinant oTau was prepared using a previously established protocol [75]. Briefly, recombinant full-length human tau protein (Tau-441, 2N4R, Cat# T-1001-2, rPeptide, Watkinsville, GA, USA) was resuspended to a final concentration of 5 µM in MES buffer (4-morpholineethanesulfonic acid hydrate, pH 6.5), and then dithiothreitol (Sigma-Aldrich Corp.) was added to a final concentration of 10 µM. This solution was incubated for 10 min at 55 °C. To induce aggregation, heparin (H19, Thermo Fisher Scientific) was subsequently added to a final concentration of 5 µM. This solution was placed on a shaking incubator set to 1000 rpm at 37 °C for 4 h to promote the formation of soluble oTau. Native gel electrophoresis followed by Western blot analysis of oTau preparations confirmed the presence of dimeric and trimeric tau species (Supplementary Figure S1), consistent with the previously published characterization [75]. Following incubation, the oTau preparation was cooled to room temperature and used immediately for neuronal treatments. Fresh oTau preparations were generated for each independent experiment. The concentration of oTau added to neuronal cultures is indicated in the corresponding figure legends.

### 2.6. Culture and Treatment of Primary Hippocampal Neurons

Culture of primary hippocampal neurons followed a previously described method [74]. In brief, hippocampal cultures were prepared from embryonic day 16 (E16) CF1 mouse fetuses. Hippocampal neurons were plated onto poly-D-lysine-coated (10 µg/ml) glass coverslips at a density of 40,000–50,000 cells per well in 12-well plates (Corning Life Sciences, Tewksbury, MA, USA). The neurons were cultured in Dulbecco’s Modified Eagle Medium/Nutrient Mixture F-12 (DMEM/F12) supplemented with 10% fetal bovine serum, 1× penicillin/streptomycin (PS), and 2 mM glutamine for 2 h, after which the medium was changed to neurobasal medium with B27 (Thermo Fisher Scientific) and 2 mM glutamine. After 21 days in vitro (DIV), murine neurons acquire an adult-like phenotype with respect to neuronal polarity, spine formation, and microtubule and neurofilament cytoskeletal architecture [76,77]. For treatments with oAβ, hippocampal neurons at 21 DIV were exposed to culture medium alone or treated with 1 µM oAβ plus CM, 0.7 nM BDNF, or 1000 nM PTX-BD4-3 (BD4-3) [74]. For the antibody blocking experiment, the same conditions were applied in the presence or absence of TrkB antibody (1:500, Cat#07-225, Upstate USA Inc., Charlottesville, VA, USA). The duration of treatment varied depending on the outcome measured. For experiments examining Fyn, pTau^Y18^ and NR2B, cells were exposed to synthetic oAβ for 20 min [41]. Tau phosphorylation was detectable after 4 h of treatment, while pre- and post-synaptic density changes, which required longer exposures, were treated for 8 h. Oligomeric tau formation indicated by T22 antibody signal was examined after 48 h. For treatments with oTau, 21 DIV hippocampal neurons were exposed to culture medium alone or treated with 50 nM oTau plus CM, 0.7 nM BDNF, or 1000 nM PTX-BD4-3 (BD4-3) for 24 h.

### 2.7. Immunofluorescence Staining, Confocal Imaging, Quantification, and Data Analysis

To analyze tau phosphorylation, oligomer formation, and other biochemical changes, immunofluorescence staining was conducted. Cells were fixed with 4% formaldehyde for 20 min, permeabilized with 80% ice-cold methanol for 6 min, and blocked with blocking buffer (3% normal donkey serum, 0.4% Triton X-100, 3% (*w*/*v*) bovine serum albumin in PBS). Cells were then incubated with primary antibodies, washed, and incubated with secondary antibodies. Primary antibodies used were rabbit polyclonal MAP2 antibody (1:1000; Cat#8707, Cell Signaling) to assess the dendrites and mouse monoclonal drebrin antibody (1:300; Cat#ADI-NBA-110, Enzo, Farmingdale, NY, USA) to assess drebrin-positive spines. To investigate tau phosphorylation and oligomer formation, mouse monoclonal anti-p-tau^Y18^ (1:300; Cat#MM-0194-P, Medimabs, Montréal, QC, Canada) and rabbit polyclonal T22 (1:500; Cat#ABN454, EMD Millipore, Burlington, MA, USA) were used. For pre- and post-synaptic density visualization, rabbit polyclonal synaptophysin antibody (1:300; Cat# ab32127, Abcam) and mouse monoclonal PSD95 antibody (1:300; Cat#200-301-G34, Thermo Fisher Scientific) were used. Other antibodies used included rabbit polyclonal anti-p-Fyn^Y416^ (1:300; Cat#2101, Cell Signaling Technology) and rabbit polyclonal anti-p-NR2B^Y1472^ (1:600; Cat#M2442, Sigma-Aldrich Corp.). Secondary antibodies were donkey anti-rabbit or anti-mouse, conjugated with either FITC or Cy3 (1:400; Jackson ImmunoResearch Laboratories, Inc., West Grove, PA, USA). All image acquisition and quantitative analyses were performed by an experimenter blinded to the treatment groups. Cultured hippocampal neurons were imaged through a Leica DM5500 confocal microscope (Leica Microsystems, Buffalo Grove, IL, USA). Fluorescence images were acquired using the LAS X software (version 3.6.1, Leica Microsystems). Laser power, gain, and offset settings were optimized using the LAS-X viewer software (Leica) to enhance signal capture while minimizing background and were maintained consistently for all treatment groups within each independent experiment. Total fluorescence pixel intensity was quantified using UN-SCAN-IT gel & graph digitizing software Ver. 6.14 (Silk Scientific Inc., Orem, UT, USA), a tool for quantifying fluorescence signal intensity from digital images, as described previously [33]. For each image, the total fluorescence intensity of the immunolabeled protein (i.e., synaptophysin, PSD95, pFyn, pNR2B, and T22) was measured. The fluorescence intensity per neuron was calculated as the total fluorescence intensity divided by the number of neurons in the field. The fluorescence intensity for each treatment group was then normalized to the mean value of the culture-medium control group, which was defined as 100%. The results were expressed as a percentage of the corresponding control. In addition, we intentionally quantified synaptophysin and PSD95 separately to independently assess pre-synaptic and post-synaptic compartments, regardless of whether a part of an actual synapse or not, as alterations in these markers do not always occur in parallel and may reflect distinct biological processes. Quantifying each marker individually allowed us to evaluate treatment effects on both pre- and post-synaptic components independently.

### 2.8. Thioflavin S Staining

Thioflavin S staining was performed and assessed using a previously established protocol [70,78]. Brains were post-fixed in 4% paraformaldehyde and sliced at 40 µm on a freezing microtome. Sections were mounted onto frosted slides and dried overnight at room temperature. Before staining, sections were permeabilized in 80% ice-cold methanol and pre-treated in 0.4% Triton X-100 for 20 min. Slides were then soaked in distilled water for 10 min, followed by 1% Thioflavin S (filtered) for 8 min. Slides were then dipped in 80% ethanol 10 times for 3 s each, soaked in 1% Thioflavin S for 8 min, and then dipped in 95% ethanol 10 times for 3 s each. Slides were then washed 3 times in double-distilled water in the dark for 5 min. Sections were coverslipped with ProLong Gold Antifade Reagent with DAPI (Thermo Fisher Scientific). Two hippocampal sections per mouse were assessed. For each hippocampal section, the entire hippocampal area was imaged and the total area of ThioS staining was quantitated using ImageJ (version 1.53).

### 2.9. Y-Maze Spontaneous Alternation Test

The Y-maze spontaneous alternation test was performed to assess behavioral performance in mice, as previously described [79]. Before testing, mice were habituated to the testing environment, a small, dimly lit room, for at least 1 h. Mice were then placed at the end of one arm of a Y-shaped maze and allowed to freely explore for 5 min. The maze consisted of three identical arms designated A, B, and C (arm A, ~8 × 5 × 3 inches; arms B and C, 6 × 5 × 3 inches; TAP Plastics, Mountain View, CA, USA). The total number of arm entries was recorded by an observer blinded to the experimental groups. A spontaneous alternation was defined as consecutive entries into all three arms without repetition and was expressed as the percentage of possible alternations and calculated as [number of alternations/(total number of arm entries − 2)] × 100 [80]. Alternate-arm return (AAR) events occur when an animal leaves an arm and moves to a new one, but then immediately returns to the starting arm, ignoring the unvisited third option (e.g., 1-2-1), and percentages were calculated as [number of AAR/(total number of arm entries − 2)]. Same-arm return (SAR) events occur when an animal exits an arm and re-enters that exact same arm in consecutive moves (e.g., 1-1, 2-2), and percentages were calculated as [number of SAR/(total number of arm entries − 1)].

### 2.10. Statistical Analyses

Statistical analyses were performed using GraphPad Prism 10.0.03 (GraphPad Software, Inc., La Jolla, CA, USA) and R (version 4.5.2) [81]. To prevent pseudoreplication and appropriately account for the hierarchical nature of our nested datasets (e.g., multiple neurons or dendritic spines analyzed per individual mouse or culture well), we employed Lognormal Linear Mixed-Effects Models (LMMs) (lme4 [82]; lmerTest [83]). Biological replicates (distinct cultures or individual mice) were designated as random intercepts, while genotype and treatment conditions were modeled as fixed effects. Main effects and interactions were estimated using the Kenward–Roger approximation for degrees of freedom, and all mixed-model findings were independently validated via 10,000-iteration parametric bootstrapping (pbkrtest [84]). For evaluating population-level structural shifts in dendritic spine density, we utilized the cumulative distribution function paired with Kolmogorov–Smirnov (KS) distributional tests in Prism.

For assays utilizing single, independent measurements per animal (such as the Western blotting assays and the Y-maze testing), 2 × 2 factorial designs were used. Normality of model residuals was assessed using the Shapiro–Wilk test. Datasets meeting normality assumptions were analyzed using two-way analysis of variance (ANOVA) with Type II sums of squares (R package: car). Datasets violating normality were analyzed using Aligned Rank Transform (ART) ANOVA [85] to accurately isolate and evaluate main effects and interactions non-parametrically. Across all models, three *a priori* planned contrasts (WT–vehicle vs. Tg–vehicle, Tg–vehicle vs. Tg–BD4-3, and WT–vehicle vs. WT–BD4-3) were explicitly evaluated using estimated marginal means or Mann–Whitney U tests. Where appropriate for comparisons, *p*-values were adjusted using the Holm–Bonferroni step-down procedure. Graphs show the mean ± SE (standard error) of at least three independent experiments.

## 3. Results

### 3.1. PTX-BD4-3 Prevents Synaptophysin and PSD95 Loss in oAβ-Exposed Hippocampal Neurons

**Figure 2:**
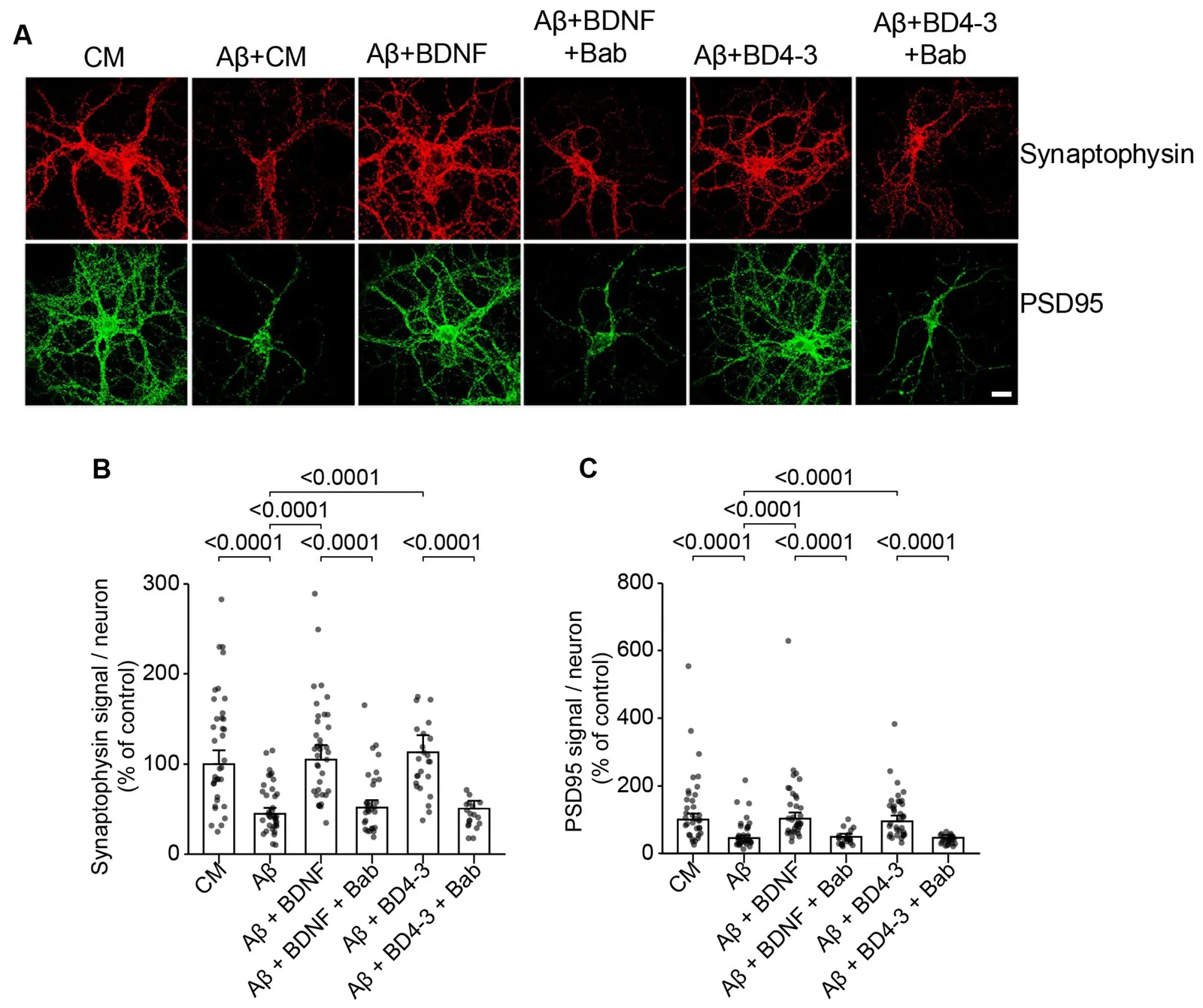
PTX-BD4-3 prevents synaptophysin and PSD95 loss in vitro. (**A**–**C**) After 21 days in vitro (DIV), hippocampal neurons were treated with culture media (CM), 1 µM oAβ, or oAβ in the presence of 1000 nM PTX-BD4-3 (BD4-3) or 0.7 nM BDNF, in the presence or absence of a TrkB-blocking antibody (Bab), and examined after 8 h. Cells were then stained with the indicated antibodies. (**A**) Representative images showing immunofluorescence staining for synaptophysin and PSD95 in hippocampal neurons. Scale bar = 10 µm. (**B**,**C**) Total fluorescence intensity was quantitated using UN-SCAN-IT software; ratios of total fluorescence intensity over total neuron number were calculated. For synaptophysin quantitation, n = 18–24 fields total, derived from 6 independent experiments. For PSD95 quantitation, n = 24–30 fields total, derived from 6 independent experiments. Statistical significance was determined using Lognormal Linear Mixed-Effects Models (LMMs) as in Materials and Methods, Section 2.10. Data are presented as mean ± SE.

In primary cultured rat hippocampal neurons, oligomeric amyloid-β (oAβ) disrupts synaptic integrity in association with reduced levels of the pre-synaptic protein synaptophysin and the post-synaptic scaffolding protein PSD95 [86], and synapse loss is observed in brains from AD patients [87] and transgenic AD mouse models [88]. To test the hypothesis that PTX-BD4-3 would have protective effects against oAβ-induced synaptic damage in vitro, we exposed hippocampal neuronal cultures prepared from E16 CF1 mice to oAβ. oAβ exposure significantly reduced the intensity of synaptophysin and PSD95 immunostaining compared to control cultures (Figure 2A–C). Co-treatment with either BDNF or PTX-BD4-3 prevented the loss of synaptophysin and PSD95, restoring their signal intensity to that observed in control cultures (Figure 2A–C). To determine whether the synaptic protective effects of PTX-BD4-3 require TrkB activation, synaptophysin and PSD95 levels were assessed following oAβ exposure in the presence of BDNF or PTX-BD4-3 and a TrkB-blocking antibody. TrkB blockade reduced the preservation of synaptophysin and PSD95 observed with either BDNF or PTX-BD4-3 treatment. These findings demonstrate that the TrkB endogenous ligand BDNF and the small-molecule TrkB partial agonist PTX-BD4-3 protect against oAβ-induced loss of synaptic proteins in a TrkB-dependent manner.

### 3.2. PTX-BD4-3 Mitigates oAβ-Induced Signaling Abnormalities and Excess Tau Phosphorylation and Oligomerization

**Figure 3:**
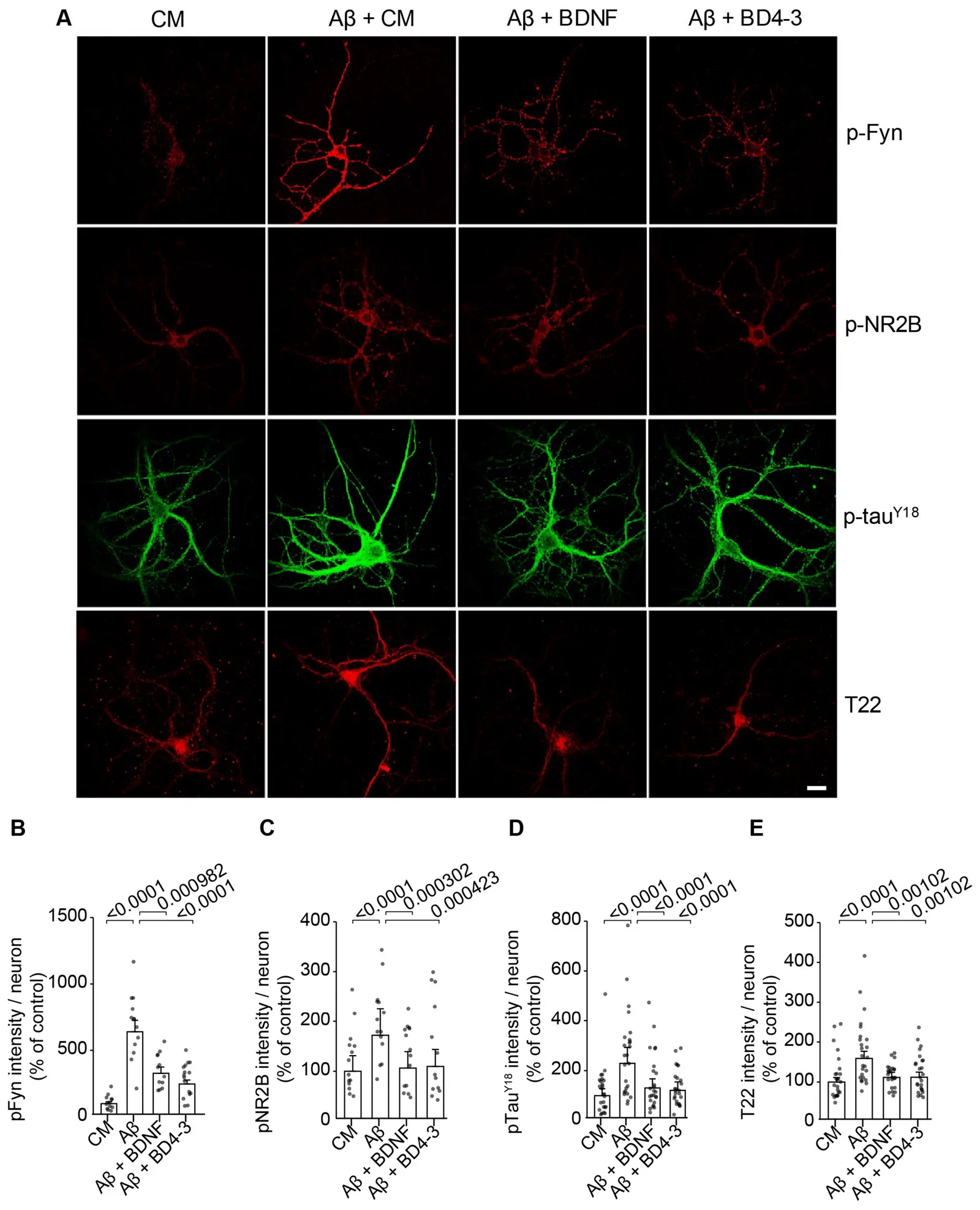
PTX-BD4-3 prevents oAβ-induced Fyn activation, NR2B and tau phosphorylation, and oligomeric tau formation. (**A**–**E**) After 21 days in vitro (DIV), hippocampal neurons were exposed to CM, 1 µM oAβ, or oAβ in the presence of 1000 nM PTX-BD4-3 or 0.7 nM BDNF. Following treatment, neurons were fixed after 20 min of treatment for Fyn and NR2B, at 4 h for pTau^Y18^, and at 48 h for T22. Cells were then immunostained with the indicated phospho-specific antibodies and a fluorophore-conjugated secondary antibody, and then imaged using immunofluorescence microscopy. Total fluorescence intensity was quantitated using UN-SCAN-IT software; ratios of total fluorescence intensity per field over total neuron number per field were calculated. (**A**) Representative immunofluorescence images. Scale bar, 10 µm. (**B**–**E**) Immunofluorescence quantitation. (**B**) For p-Fyn, n = 12–16 fields derived from 3 independent experiments. (**C**) For p-NR2B, n = 13–14 fields derived from 3 independent experiments. (**D**) For pTau^Y18^, n = 24 fields derived from 4 independent experiments. (**E**) For T22, n = 24 fields derived from 4 independent experiments. Statistical significance was determined using Lognormal Linear Mixed-Effects Models (LMMs) as in Materials and Methods, Section 2.10. Data are presented as mean ± SE.

Previous reports show that Aβ promotes Fyn activation, excess phosphorylation of the Fyn substrates NR2B and tau [33,41,89,90], and tau oligomerization [33,91], all of which contribute to synaptic dysfunction. Similarly, in hippocampal neurons exposed to oAβ, we observed elevated Fyn activation, phosphorylation of NR2B (Y1472) and tau (Y18), and tau oligomerization (Figure 3A–E). These effects were attenuated by treatment with BDNF and PTX-BD4-3 (Figure 3A–E). These findings demonstrate that PTX-BD4-3 reduces oAβ-induced excess Fyn-NR2B-tau signaling and tau oligomerization, effects that are associated with its protection against oAβ-induced loss of synaptic markers.

### 3.3. PTX-BD4-3 Inhibits Dendritic Spine Loss Induced by oTau

**Figure 4:**
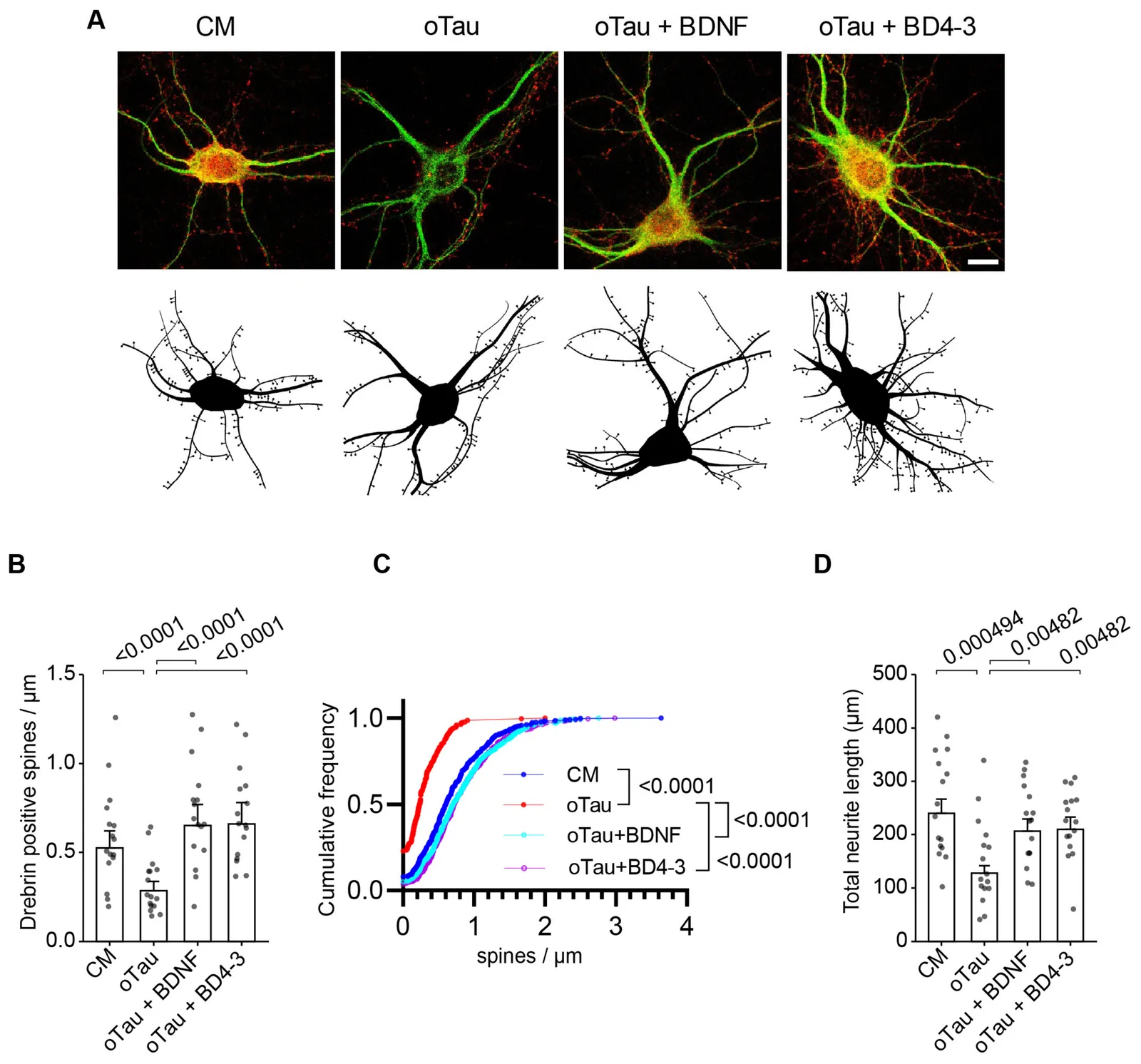
PTX-BD4-3 prevents oTau-induced spine loss in hippocampal neurons. (**A**–**D**) After 21 days in vitro (DIV), hippocampal neurons were treated with culture medium (CM), recombinant tau oligomer (oTau, 50 nM), or oTau with 0.7 nM BDNF or 1000 nM PTX-BD4-3 and then examined at 24 h following treatment. (**A**) Representative neurons stained for drebrin (red) and MAP2 (green), with drebrin-positive spines traced using MBF Bioscience Neurolucida software. Scale bar = 20 µm. (**B**) Quantitation of spine number per length of dendrite segment. (**C**) Cumulative frequency of dendritic spines/µm with *p*-values from Kolmogorov–Smirnov (KS) testing of indicated comparisons. (**D**) Total neurite length. (**B, D**) n = 16 neurons per condition from a total of 4 independent experiments for all measures. Statistical significance was determined using Lognormal Linear Mixed-Effects Models (LMMs) as in Materials and Methods, Section 2.10. Data are presented as mean ± SE.

Considering that tau is a key mediator of Aβ-driven synapse loss, and that dendritic spines form the post-synaptic compartment of most excitatory synapses, we next asked whether PTX-BD4-3 can prevent spine loss caused by oTau exposure. We exposed hippocampal neurons at 21 days in vitro (DIV) to oTau generated from recombinant tau protein, and then stained and imaged for the neuronal dendrite marker MAP2 and the dendritic spine marker drebrin. Quantitative morphological analysis revealed that recombinant oTau caused a significant ~50% decrease in spine density (Figure 4A,B), a significant uniform left shift in the cumulative frequency distribution of drebrin-positive spines/µm (Figure 4C), and a decrease in the total dendrite length (Figure 4D). Notably, PTX-BD4-3 treatment protected dendritic spines and dendrites from oTau-induced degeneration (Figure 4A–D). These results show that exposure of hippocampal neurons to recombinant oTau causes degeneration of spines and dendrites, and that this degeneration can be blocked by PTX-BD4-3.

### 3.4. PTX-BD4-3 Promotes TrkB/Akt Signaling, Inhibits Fyn/pTau^Y18^ Signaling, and Preserves Synaptic Proteins Without Affecting GFAP Expression and Amyloid Pathology in APP^L/S^ Mice

**Figure 5:**
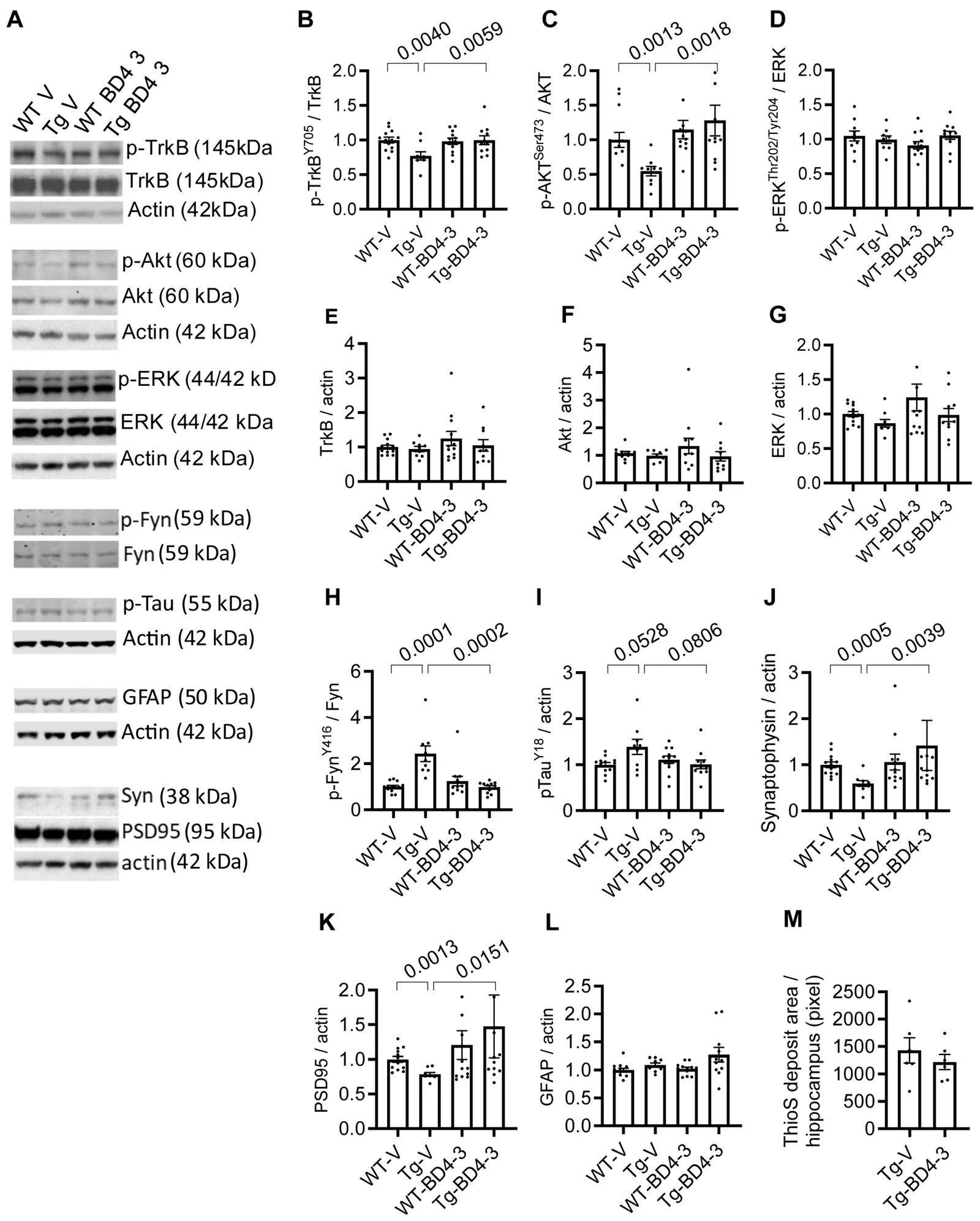
PTX-BD4-3 preserves TrkB and Akt signaling, suppresses elevated ratios of p-Fyn/Fyn and pTau^Y18^/actin, preserves synaptic proteins, and does not alter GFAP expression or amyloid burden in APP^L/S^ mice. (**A**–**M**) Five-month-old WT or APP^L/S^ (Tg) mice were treated with vehicle (V) or PTX-BD4-3 (BD4-3) for 5 days a week for 3 months. Western blots of hippocampal synaptosome extracts were quantitated by determining the ratios of phospho (p)-protein over total protein or indicated protein over actin and normalized to the control non-transgenic group (WT-V). (**A**) Representative Western blot images. (**B**) p-TrkB^Y705^/TrkB. (**C**) p-Akt/Akt. (**D**) p-ERK/ERK. (**E**) TrkB/actin. (**F**) Akt/actin. (**G**) ERK/actin. (**H**) p-Fyn/Fyn. (**I**) pTau^Y18^/actin. (**J**) Synaptophysin/actin. (**K**) PSD95/actin. (**L**) GFAP/actin. (**M**) Thioflavin S (ThioS) deposit area in average total pixel units/hippocampal section in each mouse. (**B**–**L**) Datasets meeting normality assumptions were analyzed using two-way ANOVA; datasets violating normality were analyzed using the Aligned Rank Transform ANOVA. n = 9–13 mice per group, with 2 independent Western blots averaged per animal. (**M**) ThioS staining. Statistical significance was determined using an unpaired t test; n = 7 mice per group. All data are presented as mean ± SE.

Next, we investigated the effects of PTX-BD4-3 in vivo, starting with determining whether it activates TrkB and the downstream canonical synaptoprotective pathways, PI3K/Akt and MAPK/ERK, in APP^L/S^ mice [1,2,3]. APP^L/S^ mice were treated intraperitoneally (IP) with 50 mg/kg/day PTX-BD4-3 or vehicle for 3 months starting at 5 months of age (Figure 1). Body weight was monitored weekly throughout the 3-month treatment period. APP^L/S^ mice weighed significantly less than WT mice in both age groups examined, consistent with our previously published result [92]. No treatment-related changes in body weight were observed in PTX-BD4-3-treated mice (Supplementary Figure S2A,B). Western blotting of hippocampal synaptosomes showed decreased ratios of p-TrkB^Y705^/TrkB and p-Akt^Ser473^/Akt in the hippocampus of vehicle-treated APP^L/S^ mice compared to vehicle-treated wild-type (WT) mice (Figure 5A–C, Supplementary Figure S3A,B), indicating reduced TrkB and Akt activation, respectively. Similar to previous studies in which PTX-BD4-3 increased TrkB and Akt signaling in vivo [65], treating APP^L/S^ mice with PTX-BD4-3 prevented a reduction in p-TrkB^Y705^/TrkB and p-Akt/Akt (Figure 5A–C, Supplementary Figure S3A,B). p-ERK^Thr202/Tyr204^/ERK was not altered in transgenic or PTX-BD4-3-treated groups (Figure 5D, Supplementary Figure S3C). TrkB/actin, Akt/actin, and ERK/actin were similar in WT and APP^L/S^ mice regardless of whether or not they were treated with PTX-BD4-3 (Figure 5E–G, Supplementary Figure S3A–C). These findings are consistent with activation of TrkB and Akt pathway signaling by PTX-BD4-3 in APP^L/S^ mice and are associated with the observed preservation of synapses and dendritic spines.

Given that PTX-BD4-3 suppressed oAβ-induced excess Fyn activation and pTau^Y18^, and protected against dendritic spine loss in cultured hippocampal neurons, we next investigated whether these neuroprotective effects were also observed in vivo. Consistent with our in vitro findings, PTX-BD4-3 reduced elevated ratios of pFyn/Fyn in APP^L/S^ mice (Figure 5A,H, Supplementary Figure S3D). Similarly, pTau^Y18^/actin showed a trend toward an increase in APP^L/S^ mice (*p* = 0.0528), and PTX-BD4-3 treatment showed a trend toward reducing pTau^Y18^/actin in APP^L/S^ mice (Figure 5A,I, Supplementary Figure S3E), though this reduction did not reach statistical significance (*p* = 0.0806).

We next asked whether these molecular changes were accompanied by preservation of synaptic proteins in vivo. APP^L/S^ mice exhibit amyloid pathology and a progressive loss of synaptic markers, both occurring in the frontal cortex beginning at 3–4 months of age, and in the hippocampus at 5–7 months of age (Figure 1) [68]. Similar to these previous findings, we observed a significant reduction in the ratio of synaptophysin/actin and PSD95/actin in the hippocampal extract of vehicle-treated APP^L/S^ mice compared to vehicle-treated WT mice at 8 months of age (Figure 5J,K, Supplementary Figure S3F). Treatment of APP^L/S^ mice with PTX-BD4-3 preserved the ratios of synaptophysin/actin and PSD95/actin relative to APP^L/S^ mice treated with vehicle (Figure 5A,J,K, Supplementary Figure S3F), showing that PTX-BD4-3 prevents loss of pre- and post-synaptic structural components in an in vivo AD model.

Because astrogliosis, which is associated with increased glial fibrillary acidic protein (GFAP) [93], and Aβ pathology are major contributors to synaptic dysfunction in AD [94], we also evaluated GFAP expression and Aβ plaque burden. The ratio of GFAP/actin was unchanged in APP^L/S^ mice compared to WT and was not affected by PTX-BD4-3 treatment, indicating that genotype and treatment effects observed in this study are unrelated to astrogliosis (Figure 5A,L, Supplementary Figure S3G). PTX-BD4-3 treatment also did not significantly affect Aβ plaque burden, as assessed by Thioflavin-S staining (Figure 5M), indicating that the protective effects of PTX-BD4-3 are unlikely to result from decreased amyloid deposition. In addition, actin levels did not differ significantly between the experimental groups (Supplementary Figure S3H).

### 3.5. PTX-BD4-3 Prevents Spine Loss and Restores Spine Density in APP^L/S^ Mice

Given the protective effects of PTX-BD4-3 on synaptic markers in oAβ-treated hippocampal neurons and in APP^L/S^ mice, we asked whether PTX-BD4-3 prevents spine loss or restores spine density in the hippocampus of APP^L/S^ mice across two age groups: one group with treatment initiation at 5 months followed by harvest at 8 months, a window aligning with the established onset of hippocampal synaptic degeneration in the APP^L/S^ model [68,79], and another with treatment initiation at 12 months and harvest at 15 months, representing early- and late-stage synapse loss, respectively (Figure 1) [68].

**Figure 6:**
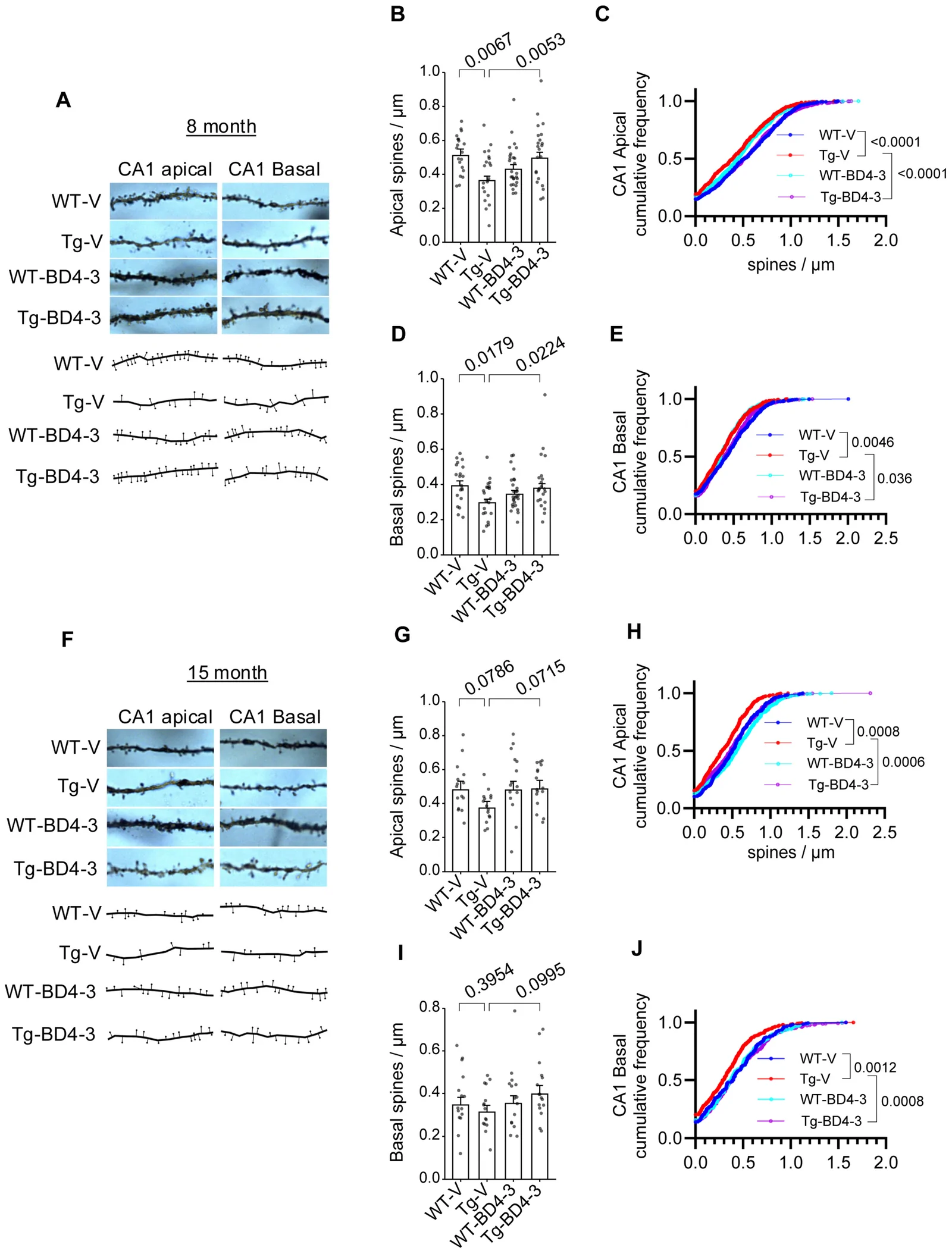
Effects of PTX-BD4-3 on spine density in the hippocampus of APP^L/S^ mice. (**A**–**J**) WT or APP^L/S^ (Tg) mice were treated with vehicle (V) or PTX-BD4-3 (BD4-3) once daily, for 5 days a week for 3 months. Hippocampal CA1 neurons from these mice were Golgi-stained, and dendrites and spines of neurons were traced using Neurolucida software. (**A**,**F**) Representative images (top eight panels) and reconstructed drawings (bottom eight panels) from Neurolucida tracings of Golgi-stained dendrites and spines from the hippocampal CA1 region of mice at 8 months or 15 months of age. (**B**–**E**) Total spine density and cumulative frequency distribution of dendritic spines/µm from mice at 8 months of age. Three neurons from each of 7–9 mice per treatment group were traced; a total of n = 21–27 neurons from each of four treatment groups were analyzed. (**B**,**C**) Apical spines. (**D**,**E**) Basal spines. (**G**–**J**) Total spine density and cumulative frequency distribution of dendritic spines/μm at 15 months of age. A total of 3–4 neurons from each of 5 mice per treatment group were traced; a total of n = 15–16 neurons from each of four treatment groups were analyzed. (**G**,**H**) Apical spines. (**I**,**J**) Basal spines. (**B**,**D**,**G**,**I**) Statistical significance for the bar graphs was determined using two-way LMMs as described in Materials and Methods, Section 2.10. Data are presented as mean ± SE. (**C**,**E**,**H**,**J**) Statistical significance for the cumulative frequency plots was determined using the Kolmogorov–Smirnov test.

Quantitative morphological analysis of Golgi-stained neurons from the younger cohort (8-month-old mice) revealed a significant decrease in average apical and basal spine density in the hippocampal CA1 region of APP^L/S^ mice compared to WT (Figure 6A,B,D).

Treatment with PTX-BD4-3 prevented the loss of apical and basal average spine density as evaluated by LMM (Figure 6A,B,D). Cumulative frequency analysis also indicated a rightward shift in the distribution of both apical and basal spines (Figure 6C,E), indicating that PTX-BD4-3 preserved dendritic spine density.

In the 15-month-old APP^L/S^ mice, LMM analysis of average spine density did not reach statistical significance for treatment effects due to elevated inter-subject variance (Figure 6F,G,I). However, cumulative frequency analysis, which evaluates the structural distribution of individual spines across the population, revealed a uniform leftward shift in apical and basal spine density distributions in APP^L/S^ animals relative to WT. Treatment of APP^L/S^ mice with PTX-BD4-3 resulted in a rightward shift in the cumulative frequency plot of spines/µm for both apical and basal spines (Figure 6H,J), indicating that PTX-BD4-3 may also restore apical and basal dendritic spine density distributions in aged APP^L/S^ mice.

Overall, in WT mice, chronic PTX-BD4-3 administration did not affect baseline signaling, dendritic spine density, or behavioral exploration. Western blot analysis across TrkB and downstream targets confirmed that PTX-BD4-3 had no effect on baseline phosphorylation or synaptic protein expression under two-way ANOVA (Figure 5; n = 12–13 per group; all *p* ≥ 0.183). For CA1 dendritic spines, two-way LMM/Kenward–Roger (KR) analysis confirmed that PTX-BD4-3 did not significantly alter WT spine density at 8 months (Figure 6B,D; apical: *p* = 0.0692; basal: *p* = 0.1543) or at 15 months (Figure 6G,I; apical: *p* = 0.6869; basal: *p* = 0.9331).

### 3.6. PTX-BD4-3 Improves Y-Maze Performance in APP^L/S^ Mice

**Figure 7:**
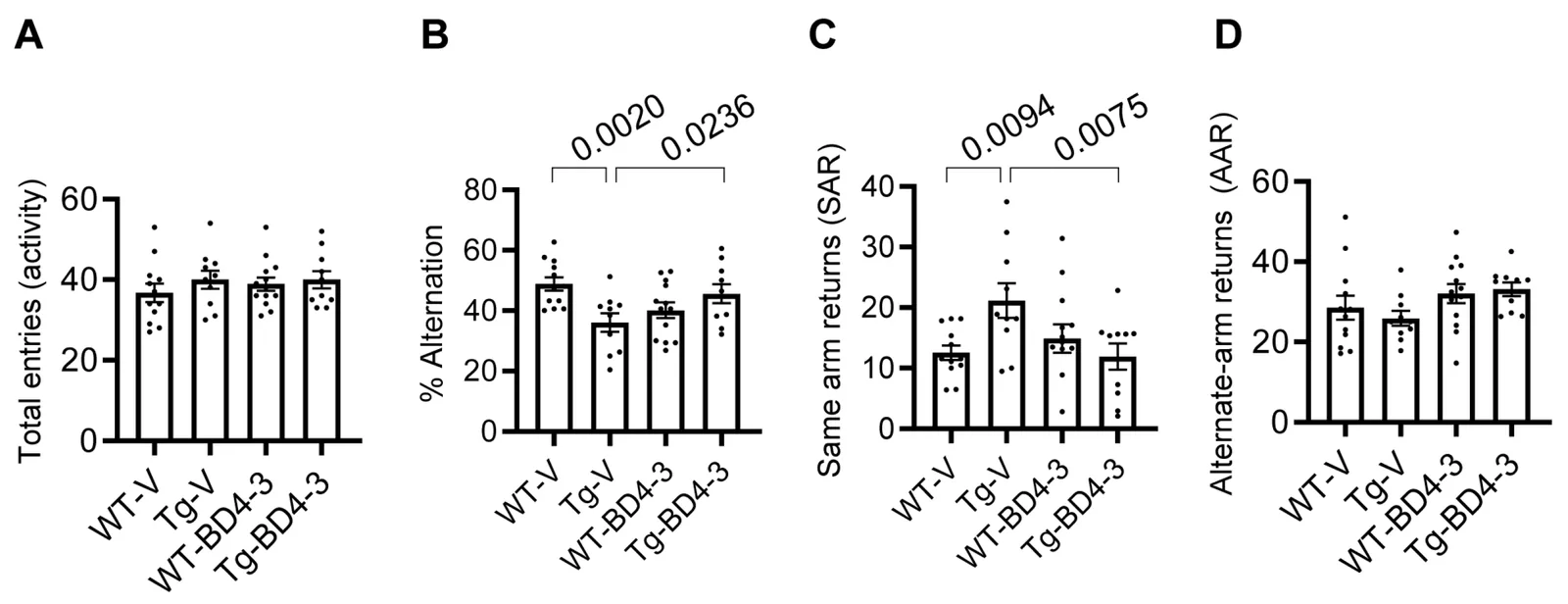
PTX-BD4-3 prevents abnormal Y-maze performance in APP^L/S^ mice. Five-month-old WT or APP^L/S^ (Tg) mice were treated with vehicle (V) or PTX-BD4-3 (BD4-3) for 5 days a week for 3 months and Y-maze testing was performed as described in Materials and Methods, Section 2.9. (**A**) Total arm entries. (**B**) Percent spontaneous alternation. (**C**) Percent same-arm returns. (**D**) Alternate-arm returns. (**A**–**D**) Statistical significance was determined by two-way ANOVA as described in Materials and Methods, Section 2.10. n = 10–13 mice per group; data are presented as mean ± SE.

Having found that PTX-BD4-3 preserves dendritic spines and synaptic proteins in vivo, we next evaluated whether these effects were associated with improved behavioral performance using the Y-maze spontaneous alternation test after 3 months of treatment, starting at 5 months of age. Total arm entries did not differ significantly among WT–vehicle, WT-PTX-BD4-3, APP^L/S^–vehicle, and APP^L/S^-PTX-BD4-3 groups (Figure 7A), indicating that locomotion and motivation to explore were unaffected by genotype or treatment. For the vehicle-treated WT mice, maze alternations were near the chance level of ~50%, demonstrating relatively poor performance, which is observed in some mouse strains, including C57BL/6-DBA/2 hybrids such as APP^L/S^ [95,96]. Compared with vehicle-treated WT mice, vehicle-treated APP^L/S^ mice exhibited a significant reduction in spontaneous alternation and an increase in same-arm returns (SAR, a measure of perseverative or compulsive behavior in which mice repeatedly re-enter the same arm [97,98]), while there was no significant change in alternate-arm returns (AAR, an intermediate perseverative behavior measured by re-entry into a previously visited arm following an intervening arm entry) (Figure 7B–D). This pattern is consistent with prior studies showing similar behavioral abnormalities in AD-model mice [99,100]. In this context, PTX-BD4-3 significantly increased spontaneous alternation in APP^L/S^ mice compared with vehicle-treated mice, restoring performance toward WT levels while normalizing SAR levels (Figure 7B–D). Collectively, these findings demonstrate that PTX-BD4-3 prevents abnormal Y-maze performance in APP^L/S^ mice. In addition, in WT mice, PTX-BD4-3 treatment significantly reduced spontaneous alternation compared with vehicle-treated WT controls (*p* = 0.0210), whereas no significant treatment effects were observed for total arm entries (*p* = 0.4409), same-arm returns (SAR; *p* = 0.4438), or alternate-arm returns (AAR; *p* = 0.2802). Because this reduction occurred in isolation without concurrent changes in locomotion or perseverative measures, its biological significance remains uncertain. Additional behavioral studies will be required to fully evaluate the effects of chronic PTX-BD4-3 administration in healthy, non-transgenic animals.

## 4. Discussion

Dendritic spine loss and synapse loss are strongly associated with cognitive decline and are well-established occurrences in AD patient brains, as well as in mouse models of AD and in in vitro models of Aβ and tau toxicity [33,77,101,102,103,104,105,106,107,108,109]. In the present study, we demonstrated that the small-molecule TrkB partial agonist PTX-BD4-3 mitigated synaptic marker and/or dendritic spine loss in cultured hippocampal neurons exposed to oAβ or oTau and in APP^L/S^ mice. Protective effects against Aβ-induced synaptic marker loss were dependent on TrkB signaling in vitro. In addition, PTX-BD4-3 prevented deficits in TrkB-Akt signaling in APP^L/S^ mice, reduced excess Fyn-NR2B-tau signaling both in vitro and in vivo, and normalized Y-maze performance in APP^L/S^ mice. These findings suggest a model in which PTX-BD4-3 promotes synaptic resilience and rebalances protective and degenerative signaling mechanisms.

Reinforcement of TrkB signaling may underlie the association of PTX-BD4-3 with synaptoprotection. Consistent with reports of reduced BDNF/TrkB signaling in AD [22,110], APP^L/S^ mice exhibit impaired TrkB activation and downstream Akt signaling. Similar to the effects of TrkB’s endogenous ligand BDNF, PTX-BD4-3 activated the TrkB receptor and downstream neuro- and synaptoprotective PI3K/Akt pathway in the hippocampus of APP^L/S^ mice, possibly contributing to preservation of spine density and synaptic integrity. However, because the final PTX-BD4-3 dose was administered one hour before tissue collection, the increases in p-TrkB and p-Akt may reflect, at least in part, an acute pharmacodynamic response and thus do not distinguish whether PTX-BD4-3 acutely activates this pathway or whether it promotes longer-term alteration of disease-impaired TrkB/Akt signaling. It has been reported that intraventricular injection of adeno-associated or lentiviral vectors encoding human BDNF activates TrkB signaling and prevents or reverses synaptic degeneration and cognitive impairment in AD-related mouse, rat, and non-human primate models [19,22]. BDNF has a short plasma half-life and does not effectively cross the BBB [44,45], making direct therapeutic administration and clinical translation challenging [1,46,47]. Various procedure-based strategies, including direct hippocampal injection of BDNF protein or BDNF-expressing vectors, enhance TrkB signaling in the brain and produce meaningful protective effects on AD phenotypes [19,20,111]. Other options for BDNF delivery have shown promise, including intravenously administered brain-targeted lipid nanoparticles, intraperitoneal delivery of membrane-penetrating BDNF fusion proteins, and intranasal delivery of native BDNF protein [112,113,114]. Another alternative for therapeutic targeting of TrkB mechanisms includes the development of small-molecule TrkB agonists that can reach the CNS and activate TrkB signaling following peripheral administration. These efforts will also provide the important contribution of expanding the pool of candidates for clinical translation.

In addition to promoting pro-survival Akt signaling, TrkB activation may counterbalance degenerative pathways engaged by Aβ or tau. Fyn was examined because it is known to interact with and influence TrkB signaling [23,24] and plays a central role in Aβ- and tau-mediated synaptotoxic signaling. Aβ oligomers induce activation of Fyn, leading to excess phosphorylation of NR2B and tau, which are associated with promotion of synaptic dysfunction [41,43]. Consistent with this counterbalancing mechanism, PTX-BD4-3 reduced Fyn activation and downstream phosphorylation of tau and NR2B, in association with its synaptoprotective effects. Targeting this signaling is supported by prior studies showing that Fyn depletion [115,116] and tau knockout [28] can protect synapses from Aβ-induced excitotoxic degeneration. Additionally, excess phosphorylation of NR2B at Y1472 promotes surface expression and activation of extrasynaptic NMDARs [26,117,118], which can oppose the neuroprotective effects of synaptic NMDARs. By reducing NR2B phosphorylation, PTX-BD4-3 may limit this shift toward excitotoxic signaling. Thus, PTX-BD4-3 influences multiple signaling pathways associated with enhanced synaptic resilience.

PTX-BD4-3 also protected against oTau-induced dendritic spine loss in hippocampal neurons, and the ability of TrkB signaling to protect neurons from both oAβ- and oTau-mediated toxicity may offer advantages over therapeutic strategies that target only oAβ or oTau. Furthermore, combining FDA-approved Aβ-lowering therapies with agents activating TrkB may enhance therapeutic efficacy through additive or synergistic effects [119,120,121]. Beyond the TrkB-associated signaling pathways examined here, PTX-BD4-3 may promote synaptic integrity through additional downstream mechanisms, including modulation of oxidative stress or regulation of cytoskeletal integrity, which could be explored in future studies.

While data presented in the present and prior studies support a model in which PTX-BD4-3 functions by activation of TrkB, it remains possible that the compound functions through one or more other and/or additional targets. Whether PTX-BD4-3 directly or indirectly activates TrkB signaling remains to be fully established. Future structure–activity-relationship studies will be important for the full interrogation of the TrkB target.

The question of function through TrkB is also relevant to the parent compound LM22A-4. Two studies employing cell lines in vitro failed to detect activation of TrkB [122,123], whereas another study suggested that its ability to activate TrkB resulted from transactivation [124]. In contrast, numerous studies have demonstrated that LM22A-4 activates TrkB and its associated signaling pathways and/or produces biological effects that are dependent on TrkB in vitro or in vivo, in a variety of tissues and cell types from mouse and rat models [65,125,126,127,128,129,130,131,132,133,134,135,136,137,138,139,140]. Overall, these studies suggest the possibility that the function of small-molecule TrkB modulators might be context-dependent.

Several limitations of this study should be considered. The APP^L/S^ mouse model primarily recapitulates Aβ pathology and early-stage tau pathology [141], but does not capture the full spectrum of tau-related neurodegeneration, such as accumulation of paired helical filaments, that occurs in human AD. Additionally, the treatment duration was limited to 3 months, and long-term effects remain to be explored. Furthermore, we did not assess the effects of PTX-BD4-3 on non-hippocampal regions. Our in vitro experiments utilized recombinant oAβ and oTau, which may not fully reflect endogenous pathological protein states. Although our TrkB-blocking experiments demonstrate that PTX-BD4-3-mediated preservation of synaptophysin and PSD95 following oAβ exposure is TrkB-dependent, we did not demonstrate TrkB dependence for the effects of PTX-BD4-3 on Fyn/NR2B/tau signaling, tau oligomerization, oTau-induced dendritic spine loss, or the in vivo outcomes. Future studies employing pathway-specific inhibition or genetic approaches will be required to determine which of these molecular events are causally linked to the synaptoprotective effects of PTX-BD4-3. Finally, while our findings show beneficial effects on signaling and on synapses, further studies are needed to evaluate drug effects on functional outcomes such as long-term potentiation and behavior.

## 5. Conclusions

Activation of TrkB signaling by PTX-BD4-3 prevented synapse loss and was associated with decreased oAβ- and oTau-mediated dendritic spine loss, and normalization of oAβ-induced changes in Fyn-NR2B-tau signaling in vitro. In APP^L/S^ mice, PTX-BD4-3 prevented deficits in TrkB/Akt signaling, reduced excess Fyn and tau phosphorylation, preserved synaptic protein markers, restored dendritic spine density, and improved Y-maze performance. These results suggest that small molecules activating TrkB may be promising candidates for AD therapeutic development as well as valuable tool compounds for manipulating and studying TrkB signaling mechanisms of synaptic resilience. Further research is required to determine whether PTX-BD4-3 can alleviate synaptic impairment and cognitive decline in both amyloid- and tau-based models.

## Data Availability

The datasets generated during and/or analyzed during the current study are available from the corresponding authors on reasonable request.

## Supplementary Materials

The following supporting information can be downloaded at: https://www.mdpi.com/article/10.3390/cells15191822/s1, Supplementary Figure S1: Native gel electrophoresis followed by immunoblot analysis using the total tau antibody HT7 demonstrated that the tau oligomer preparation contained multiple tau species, including dimers and trimers, consistent with previously published findings [75]; Supplementary Figure S2. Body weights were tracked weekly across 13 weeks of dosing in both young (5–8-month) (A) and aged (12–15-month) (B) cohorts. Data were analyzed using a 3-Way Linear Mixed-Effects Model with repeated measures. APP^L/S^ mice weighed significantly less than WT mice in both age groups examined, consistent with our previously published result [92]. In the young cohort, all animals exhibited normal developmental weight gain over time (F(2.609, 111.3) = 33.23, *p* < 0.0001), with no significant interaction between time and treatment group (F(7.826, 111.3) = 1.235, *p* = 0.2860). Similarly, in the aged cohort, body weights remained stable throughout the dosing period with no time-by-group interaction (F(10.83, 148.9) = 1.224, *p* = 0.2765). Comparisons of overall body weight revealed no significant treatment effects of the compound versus vehicle controls in wild-type mice (Aged: *p* = 0.848; Young: *p* = 0.921) or transgenic mice (Aged: *p* = 0.914; Young: *p* = 0.991), confirming absence of compound-mediated weight effects; Supplementary Figure S3: (A–G) Uncropped, representative western blot images corresponding to cropped images shown in Figure 5: (A) p-TrkBY705, TrkB, and actin, (B) p-Akt, Akt, and actin, (C) p-ERK, ERK, and actin, (D) p-Fyn and Fyn, (E) pTauY18 and actin, (F) synaptophysin (Syn) and postsynaptic scaffolding protein (PSD95), (G) GFAP and actin. (H) Actin levels across all experimental conditions were quantitated and normalized to the control WT group (WT-V). Statistical significance was determined using ANOVA with Dunnett’s post-hoc test; data are presented as mean ± SE, n = 9–13 mice per group.

## Abbreviations

7,8-DHF	7,8-dihydroxyflavone.
AAR	alternate-arm return.
Aβ	amyloid beta.
AD	Alzheimer’s disease.
Akt	protein kinase B.
ANOVA	analysis of variance.
APP^L/S^ mice	APP London/Swedish mice.
APP	amyloid precursor protein.
Bab	TrkB-blocking antibody.
BDNF	Brain-Derived Neurotrophic Factor.
BBB	blood–brain barrier.
CM	culture media.
CNS	Central Nervous System.
DIV	days in vitro.
DMEM/F12	Dulbecco’s Modified Eagle Medium/Nutrient Mixture F-12.
DMSO	dimethyl sulfoxide.
DPX	distyrene–plasticizer–xylene mounting medium.
ERK	extracellular signal-regulated kinase.
GFAP	glial fibrillary acidic protein.
HRP	horseradish peroxidase.
IP	intraperitoneal.
KS	Kolmogorov–Smirnov.
LMM	lognormal linear mixed-effects model.
MAP2	microtubule-associated protein 2.
MAPK	mitogen-activated protein kinase.
MES	2-(N-morpholino) ethanesulfonic acid.
NMDA	N-Methyl-D-Aspartate.
NMDAR	N-Methyl-D-Aspartate Receptor.
NR2B	subunit 2B of the NMDA receptor.
oAβ	oligomeric amyloid beta.
oTau	oligomeric tau.
p-Akt	phosphorylated Akt.
p-ERK	phosphorylated ERK.
p-Fyn	phosphorylated Fyn.
p-NR2B^Y1472^	NMDAR-NR2B phosphorylated at Y1472.
pTau^Y18^	tau phosphorylated at Tyrosine 18.
p-TrkB^Y705^	TrkB phosphorylated at Tyrosine 705.
PBS	phosphate-buffered saline.
PI3K	phosphoinositide 3-kinase.
PKC	protein kinase C.
PLCγ	phospholipase C gamma.
PSD95	post-synaptic density protein 95.
SAR	same-arm return.
SE	standard error.
Tg	transgenic.
ThioS	Thioflavin S.
TrkB	tropomyosin receptor kinase B.
V	vehicle.
WT	wild-type.
