# Supplementary material for: A Small-Molecule TrkB Agonist Promotes Synaptic Resilience Against Aβ and Tau Oligomers and Mitigates Alterations in TrkB-Akt and Fyn Signaling
Source: Cells. 2026 Oct 7;15(19):1822. doi: 10.3390/cells15191822 (PMC13664222; doi:10.3390/cells15191822)
Supplement: Supplementary file 1 [file cells-15-01822-s001.zip › cells-4534481-supplementary.pdf]

Figure S1

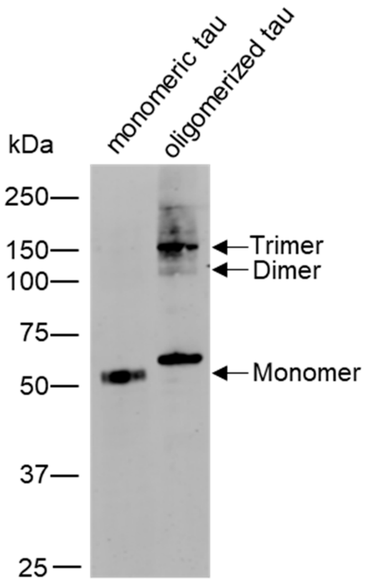

Supplementary Figure S1: Native gel electrophoresis followed by immunoblot analysis using the total tau antibody HT7 demonstrated that the tau oligomer preparation contained multiple tau species, including dimers and trimers, consistent with previously published findings [75].

Figure S2

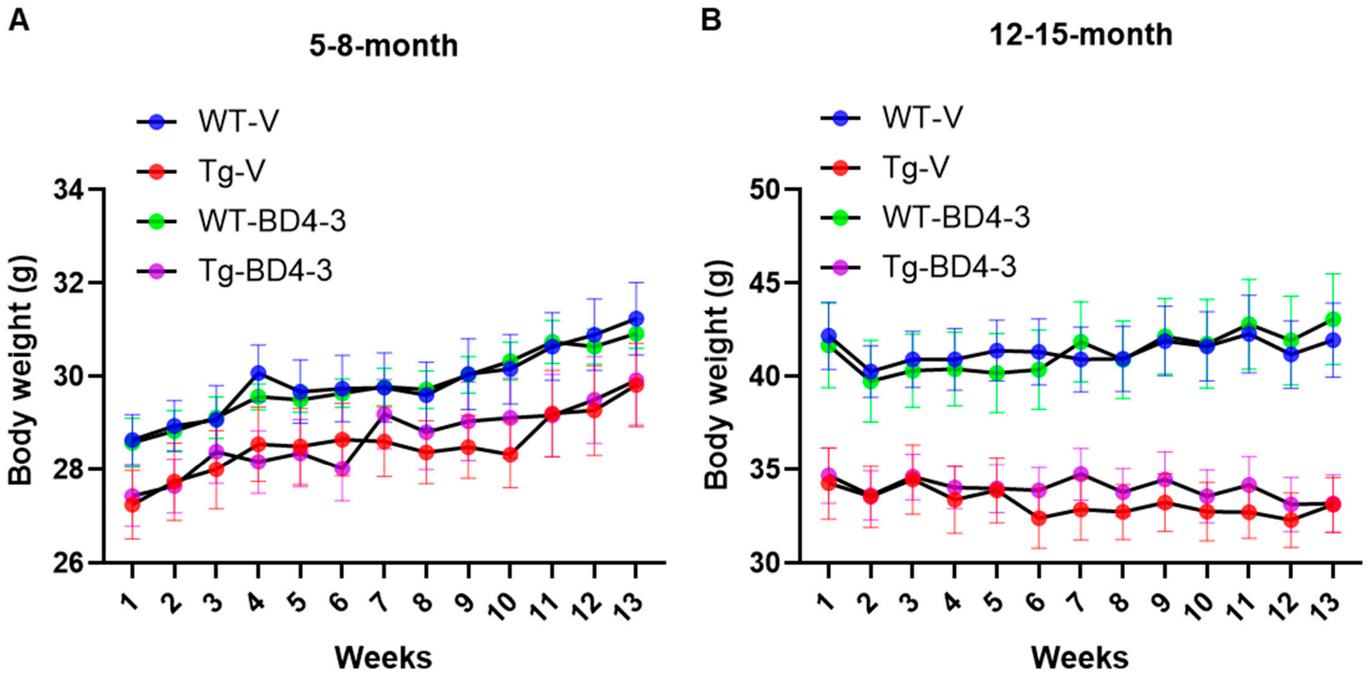

Supplementary Figure S2. Body weights were tracked weekly across 13 weeks of dosing in both young (5-8-month) (**A**) and aged (12-15-month) (**B**) cohorts. Data were analyzed using a 3-Way Linear Mixed-Effects Model with repeated measures. $APP^{L/S}$ mice weighed significantly less than WT mice in both age groups examined, consistent with our previously published result [92]. In the young cohort, all animals exhibited normal developmental weight gain over time ($F(2.609, 111.3) = 33.23$, $p < 0.0001$), with no significant interaction between time and treatment group ($F(7.826, 111.3) = 1.235$, $p = 0.2860$). Similarly, in the aged cohort, body weights remained stable throughout the dosing period with no time-by-group interaction ($F(10.83, 148.9) = 1.224$, $p = 0.2765$). Comparisons of overall body weight revealed no significant treatment effects of the compound versus vehicle controls in wild-type mice (Aged: $p = 0.848$; Young: $p = 0.921$) or transgenic mice (Aged: $p = 0.914$; Young: $p = 0.991$), confirming absence of compound-mediated weight effects.

Figure S3

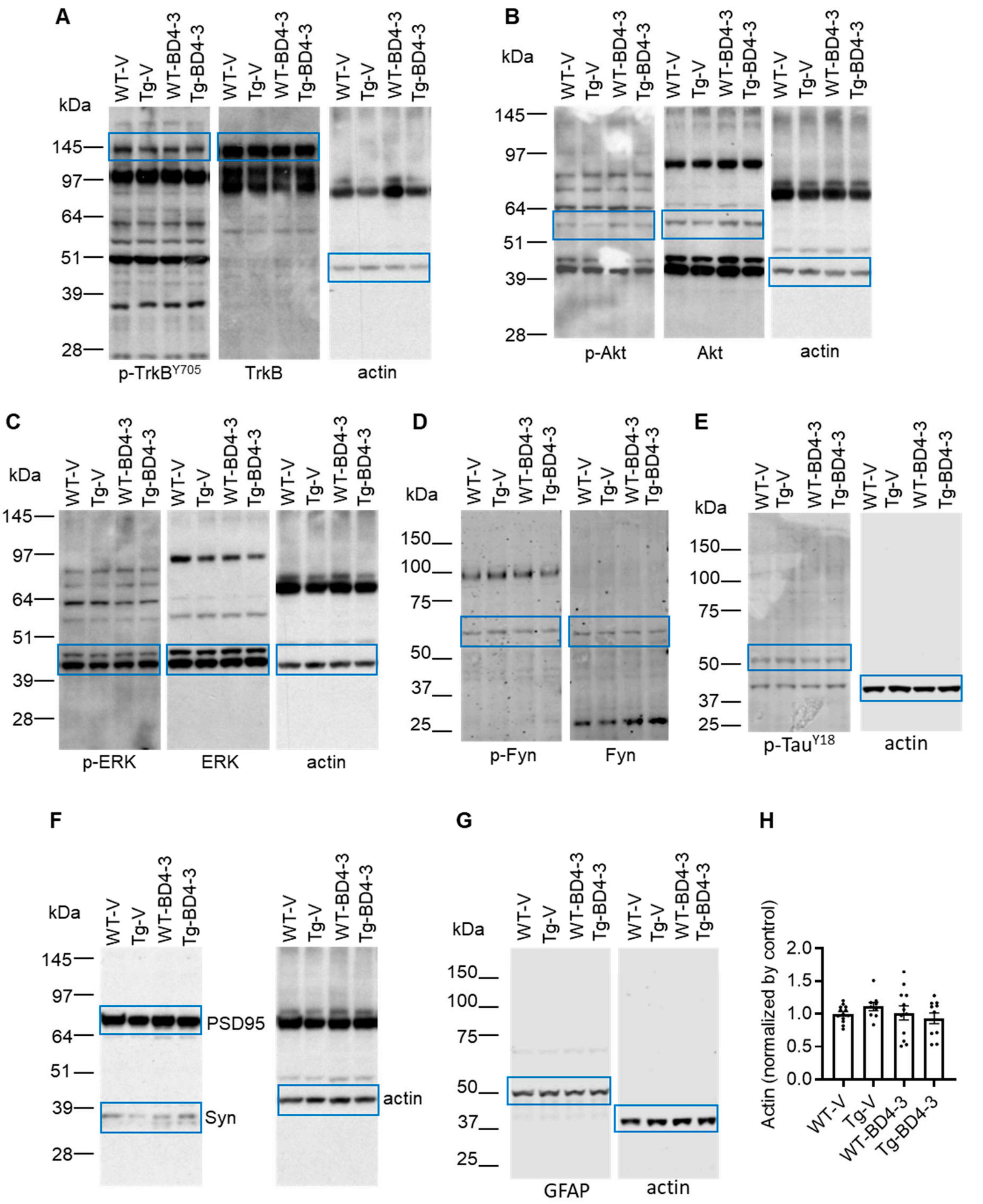

Supplementary Figure S3: (A–G) Uncropped, representative western blot images corresponding to cropped images shown in Figure 5: (**A**) p-TrkB^{Y705}, TrkB, and actin, (**B**) p-Akt, Akt, and actin, (**C**) p-ERK, ERK, and actin, (**D**) p-Fyn and Fyn, (**E**) pTau^{Y18} and actin, (**F**) synaptophysin (Syn) and postsynaptic scaffolding protein (PSD95), (**G**) GFAP and actin. (**H**) Actin levels across all experimental conditions were quantitated and

normalized to the control WT group (WT-V). Statistical significance was determined using ANOVA with Dunnett's post-hoc test; data are presented as mean ± SE, n = 9–13 mice per group.
